# Navigating through uncertainty—Experience from the UK national VEXAS MDT

**DOI:** 10.1111/bjh.70365

**Published:** 2026-02-24

**Authors:** Daniel Pietsch, Austin Kulasekararaj, Sinisa Savic, VEXNET‐UK MDT, Adam Al‐Hakim, Tanya Basu, Catherine Cargo, Onima Chowdhry, James Galloway, Eiphyu Htut, Stephen Jolles, Arvind Kaul, Helen Lachmann, Calman A. MacLennan, Anoop Mistry, Elspeth Payne, James Poulter, Farzana Rahman, Manoj Raghavan, Rachel Tattersall, Roochi Trikha, Villyn Yong, Taryn Youngstein, Anna Babb, Celia Beynon, Sarah Bingham, Nuno Borges, Jenny Bosworth, Carlos Campani, Marian Chan, Shikha Chattree, Niall Conlon, Robert Corser, Elena Ganendra, Paraskevi Gkreka, William Gordon, Elisabeth Grey‐Davies, Joanna Haughton, Simona Huica, Pawel Kaczmarek, Alison Laing, Areti Makrygeorgou, Susanna Mathew, Jill Mccormick, Muhammad Mohsin, Vidhya Murthy, Sateesh Nagumantry, Ognjenka Savanovic‐Abel, Shaun Smale, Alex Sternberg, Rosemary Waller, Sarah Westbury

**Affiliations:** ^1^ Department of Clinical Immunology and Allergy St James's University Hospital Leeds UK; ^2^ Division of Rheumatology and Clinical Immunology Medical University of Graz Graz Austria; ^3^ Haematology Department King's College Hospital NHS Foundation Trust London UK; ^4^ Leeds Institute of Rheumatic and Musculoskeletal Medicine University of Leeds Leeds UK; ^5^ NIHR Leeds Biomedical Research Centre Leeds UK

**Keywords:** azacitidine, corticosteroids, haematology, multidisciplinary team, rheumatology, UBA1, VEXAS syndrome

## Abstract

The objective of this study was to describe the establishment, structure and influence of the United Kingdom national multidisciplinary team (MDT) for vacuoles, E1 enzyme, X‐linked, autoinflammatory, somatic (VEXAS) syndrome and to assess its clinical outputs and perceived value among participating clinicians. All patients discussed at the national VEXAS MDT between June 2024 and May 2025 were included. Clinical information was extracted from standardised referral forms, and MDT recommendations were reviewed. An anonymised questionnaire evaluated clinicians' experience of the MDT. Forty‐two patients from 27 centres were reviewed; 36 (85.7%) had confirmed VEXAS syndrome and 6 had VEXAS‐like disease. Almost all were male (41/42), with a median age of 70 years. Before MDT review, 62% received corticosteroid monotherapy. MDT recommendations favoured steroid‐sparing strategies, with marked increases in the use of azacitidine (1–22 patients) and tocilizumab (5–15 patients), alongside reduced use of conventional disease‐modifying anti‐rheumatic drugs (DMARDs). Four patients were referred for haematopoietic stem cell transplantation. Twenty of 43 MDT members completed the survey, reporting greater confidence in managing VEXAS and valuing the MDT's educational and collaborative benefits. The national VEXAS MDT supported evidence‐informed, multidisciplinary decision‐making, promoted more targeted therapy and strengthened clinical confidence and collaboration in the absence of formal treatment guidelines.

## INTRODUCTION

Five years have now passed since the initial description of VEXAS (vacuoles, E1 enzyme, X‐linked, autoinflammatory, somatic) syndrome, a systemic autoinflammatory disorder resulting from somatic mutations in the UBA1 gene.^1^ VEXAS syndrome is a complex disease characterised by diverse manifestations driven by systemic inflammation—such as recurrent fever, chondritis and neutrophilic skin rashes—and bone marrow dysfunction, including myelodysplastic syndrome (MDS) and multifactorial anaemia, which often results in transfusion dependency.^2^, ^3^, ^4^ As a result, diagnosis and management typically require a multidisciplinary approach involving several specialties.

As with many newly recognised diseases, it takes time to establish international collaborative networks and develop management guidelines. For VEXAS syndrome, a guidance statement has only recently been published.^5^ In clinical practice, most patients respond well to corticosteroids, which typically control the inflammatory manifestations and can also improve anaemia, at least initially. However, long‐term steroid use often leads to significant toxicity, making steroid‐sparing strategies essential. To date, various therapeutic approaches have been explored, including anti‐cytokine agents such as tocilizumab, JAK inhibitors like ruxolitinib and clone‐directed therapies such as azacitidine, commonly used in myelodysplastic syndromes.^6^, ^7^ These treatments have shown variable success. In selected cases, haematopoietic stem cell transplantation (HSCT) has also been applied, offering a potential curative option, although experience remains limited, and with potential for serious complication still remaining a concern.^8^

In the absence of evidence‐based standards, management remains highly individualised, reflecting both the clinical heterogeneity of VEXAS syndrome and gaps in our understanding of its pathogenesis. Although research is beginning to clarify the disease mechanisms, treatment decisions continue to be complex and must be tailored to the individual patient. Furthermore, patients often initially present to a single speciality—most commonly Rheumatology, Haematology or Dermatology—yet their work‐up and management typically require coordinated input from multiple specialties.

To help address these clinical challenges, a multidisciplinary team (MDT) meeting was established in the United Kingdom with participation from Ireland, bringing together experts from haematology, rheumatology, immunology and dermatology. The primary purpose of this MDT is to provide case‐based discussions and specialist recommendations for individual VEXAS patients, supporting treating physicians in navigating complex management decisions. Participants are encouraged to provide updates on outcomes of previously discussed cases and rediscuss cases when necessary. The meetings have been held monthly via video conference since June 2024 and are open to any clinician seeking advice on the diagnosis or treatment of affected individuals.

In this report, we describe the structure and process of the MDT, summarise the cases reviewed and recommendations made and share feedback from participating clinicians. Our aim is to provide insight into the value of a collaborative, cross‐speciality approach to managing VEXAS syndrome.

## METHODS

### National VEXAS MDT

This MDT is an initiative of the UK VEXAS Interest Group (VEXNET) and is composed of a chairperson and co‐chairperson—senior physicians specialising in haematology and immunology—along with a panel of experienced haematologists, rheumatologists, dermatologists and immunologists and a case presenter. To present a case at the MDT meeting, the presenter or referrer must complete a standardised referral form detailing the disease history, as well as including relevant imaging, pathology and laboratory results (Figure S1). Cases are presented in a structured manner following the referral form, followed by a guided discussion by the panel. The chair and co‐chair then summarise the discussion and formulate outcome recommendations. Recommendations were consensus‐based rather than determined by formal voting or guidelines. When multiple therapeutic approaches were deemed suitable, the MDT discussed advantages and disadvantages of each option, leaving the final decision to the treating physician.

Most of the patients presented are VEXAS patients. However, the MDT can discuss other patients with VEXAS‐like conditions, such as certain MDS patients with inflammatory complications.

The MDTs take place monthly via video call, so people all over the country and even across national borders can participate. Each case is presented in a structured manner as laid out by the referral form.

### Eligibility and case selection

All patients who were discussed at the VEXAS MDT between June 2024 and May 2025 were included in this study.

### Clinical and phenotypic data collection

The data were collated from standardised referral sheets and further processed using Microsoft Excel (Microsoft Corporation, Redmond, WA, USA) and IBM SPSS Statistics for Windows, Version 29.0 (IBM Corp., Armonk, NY, USA).

### MDT evaluation—questionnaire

A standardised and anonymous survey was sent out to all participants of the MDT who either joined as a case presenter or as part of the panel. The survey was conducted via Google forms, assessed the subjective perception of the usefulness of the MDT and gave the opportunity for feedback.

### Ethical considerations

Ethics approval was waived since this work was done as part of a locally approved audit project using only routinely collected and fully anonymised data.

### Statistical analysis

The statistical analyses were conducted using Excel 365 and IBM SPSS 29. Median and interquartile range (IQR) were used accordingly. Figures were created using R version 4.5.1 (R Foundation for Statistical Computing) with the ggplot2 package. Only data from first‐time referrals were used for analysis.

## RESULTS

During a 1‐year period, 42 different patients from 27 centres across the United Kingdom and Ireland were discussed at the monthly national VEXAS MDT meeting. Three of those patients were discussed at multiple MDT meetings.

### Patient's characteristics

Almost all patients were male (41/42) and the median age was 70 years. The vast majority were VEXAS patients (36/42, 85.7%), although a remarkable number of patients with VEXAS‐like conditions were also discussed (6/42, 14.3%). The six patients categorised as VEXAS‐like syndrome (Table S1) represented a heterogeneous group with overlapping clinical features but lacking pathogenic *UBA1* mutations. This included patients with MDS and prominent inflammatory manifestations, as well as patients with inflammatory syndromes and unexplained cytopenias where VEXAS was considered in the differential diagnosis. These cases were discussed to aid diagnostic clarification and management guidance. Over 80% of confirmed VEXAS patients were diagnosed with a canonical mutation at the methionine 41 position of the UBA1 gene, with a median variant allele frequency of 74% (IQR 61%–80%).

Almost half of the patients (19/42, 45.2%) were transfusion‐dependent, and almost all patients were taking corticosteroids at a median daily dose of 15 mg prednisolone equivalent. Over 60% of patients were on steroids as their only therapy at the time of referral to the MDT.

Table 1 provides a summary of the baseline patients' main characteristics.

**TABLE 1:** Patient's baseline characteristics.

Characteristics	*N*/median [IQR]	Rate, % or *n* (if variable)
Number of patients	42	
Age	70 [65–79]	
Sex—male	41	97.6%
Diagnosis		
VEXAS	36	85.7%
VEXAS‐like syndrome	6	14.3%
Variant (in VEXAS patients)		
Met41Thr	12	33.3%
Met41Leu	12	33.3%
Met41Val	7	19.4%
Other pathogenic variant	4	11.1%
Missing	1	2.8%
Variant allele frequency	74% [61.0–80.0%]	
Laboratory features		
Haemoglobin (g/dL)	94.50 [79–112]	42
White cell count (10^9^/L)	5.20 [2.97–6.85]	40
Neutrophils (10^9^/L)	3.39 [2.10–5.00]	38
Lymphocytes (10^9^/L)	0.70 [0.50–1.10]	37
Platelets (10^9^/L)	119.5 [72–172]	42
CRP (mg/L)	32.5 [10–61]	36
Clinical features		
Fatigue	38	90.5%
Fever	18	42.9%
Rash	26	61.9%
(Poly‐)chondritis	18	42.9%
Arthritis/arthralgia	21	50%
Vasculitis	6	14.3%
Venous thromboembolism	14	33.3%
Respiratory symptoms	18	42.9%
Transfusion dependency	19	45.2%
Treatment before MDT		
Steroids	39	92.9%
Steroid dose (prednisolone equivalent in mg per day)	15 [8–30]	
Steroids only	26	61.9%
Azacitidine	1	2.4%
Tocilizumab	5	11.9%
Anakinra	1	2.4%
JAKi	4	9.5%
Methotrexate	6	14.3%
Other csDMARD	8	19%
Other bDMARD	2	4.8%

*Note*: Other conventional synthetic disease‐modifying anti‐rheumatic drugs (csDMARDs) include methotrexate, sulfasalzine, mycophenolate mofetil, azathioprine, leflunomide; other biological (b) DMARDs include infliximab and rituximab; other UBA1 variants include p.Ala478Ser, p.Ser56Phe, p.Ser621Cys, splice site variant c.118‐1G>C. Values are given as median (IQR: interquartile range).

Abbreviations: CRP, C‐reactive protein; MDT, multidisciplinary team; VEXAS, vacuoles, E1 enzyme, X‐linked, autoinflammatory, somatic.

### MDT outcomes and recommendations

**FIGURE 1:**
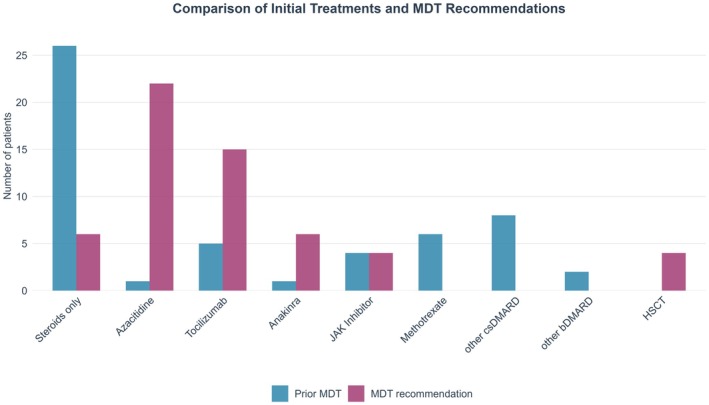
Comparison of initial treatments and multidisciplinary team (MDT) recommendations.

The data reveal a substantial reduction in the use of steroids alone, from 26 patients before the MDT to seven patients after. Conversely, recommendations for azacitidine and tocilizumab increased notably, from 1 to 22 and 5 to 15 patients respectively. Similarly, anakinra use was recommended more frequently post‐MDT (6 vs. 1). Treatments such as methotrexate, other csDMARDs and other bDMARDs were discontinued or not recommended following the MDT review. The number of patients recommended for haematopoietic stem cell transplantation rose from none to four. Those patients were younger than the average (44–64 years old) and transfusion independent. The recommendation for the use of JAK inhibitors remained stable (see Figure 1).

In some cases, more than one therapeutic recommendation was made for the same patient. This reflects both the clinical heterogeneity of VEXAS and the individual expertise of the clinician and pragmatic considerations, such as varying drug availability across different centres. Where more than one treatment approach was considered appropriate, the MDT considered the advantages and disadvantages of each option, leaving the final decision to the treating physician based on accessibility, prior therapies and individual patient factors.

In a few cases (*n* = 4), no treatment recommendation was given, as the outcome was either a recommendation for further investigations or pre‐existing patient preferences such as best supportive care (not shown).

### MDT survey results

Twenty of 43 participants, panel members as well as case presenters, returned the questionnaire in time and were included in this survey. Some participants did not answer all individual items; therefore, response rates may vary slightly between questions.

**FIGURE 2:**
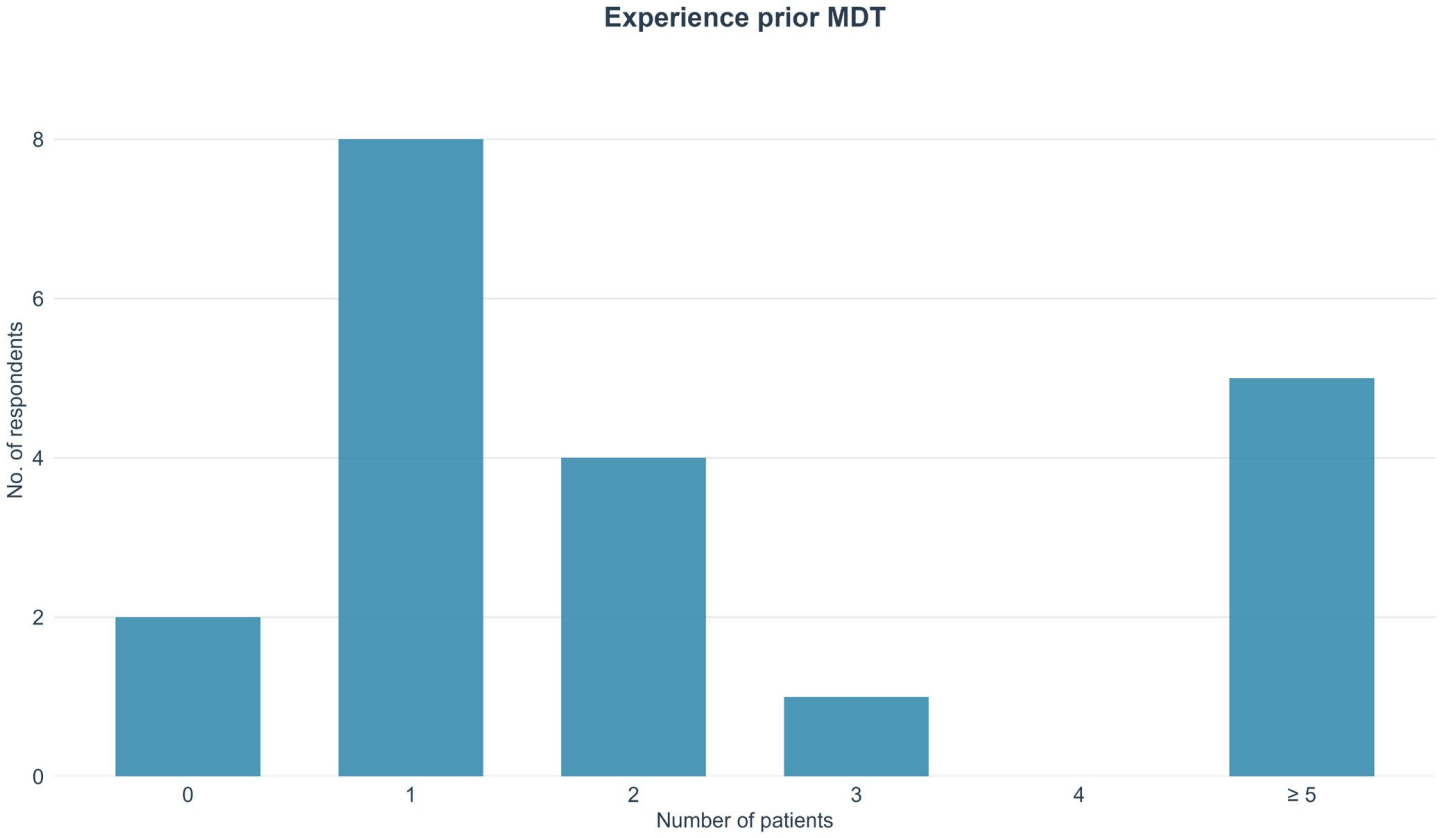
Participants' prior experience with vacuoles, E1 enzyme, X‐linked, autoinflammatory, somatic (VEXAS) syndrome before the multidisciplinary team (MDT). The *x*‐axis shows the number of VEXAS patients each respondent had been involved with, while the *y*‐axis represents the number of survey respondents in each category.

Most participants providing feedback were haematologists (*n* = 11), followed by rheumatologists (*n* = 5) and immunologists and dermatologists (*n* = 2 each). One quarter stated that they have been involved in the treatment of more than five VEXAS patients prior to the MDT, but the majority had experience with only one or two patients (Figure 2).

Participants reported low to moderate confidence in managing VEXAS syndrome prior to attending the MDT. This is consistent with the generally limited prior experience of VEXAS syndrome among participants.

**FIGURE 3:**
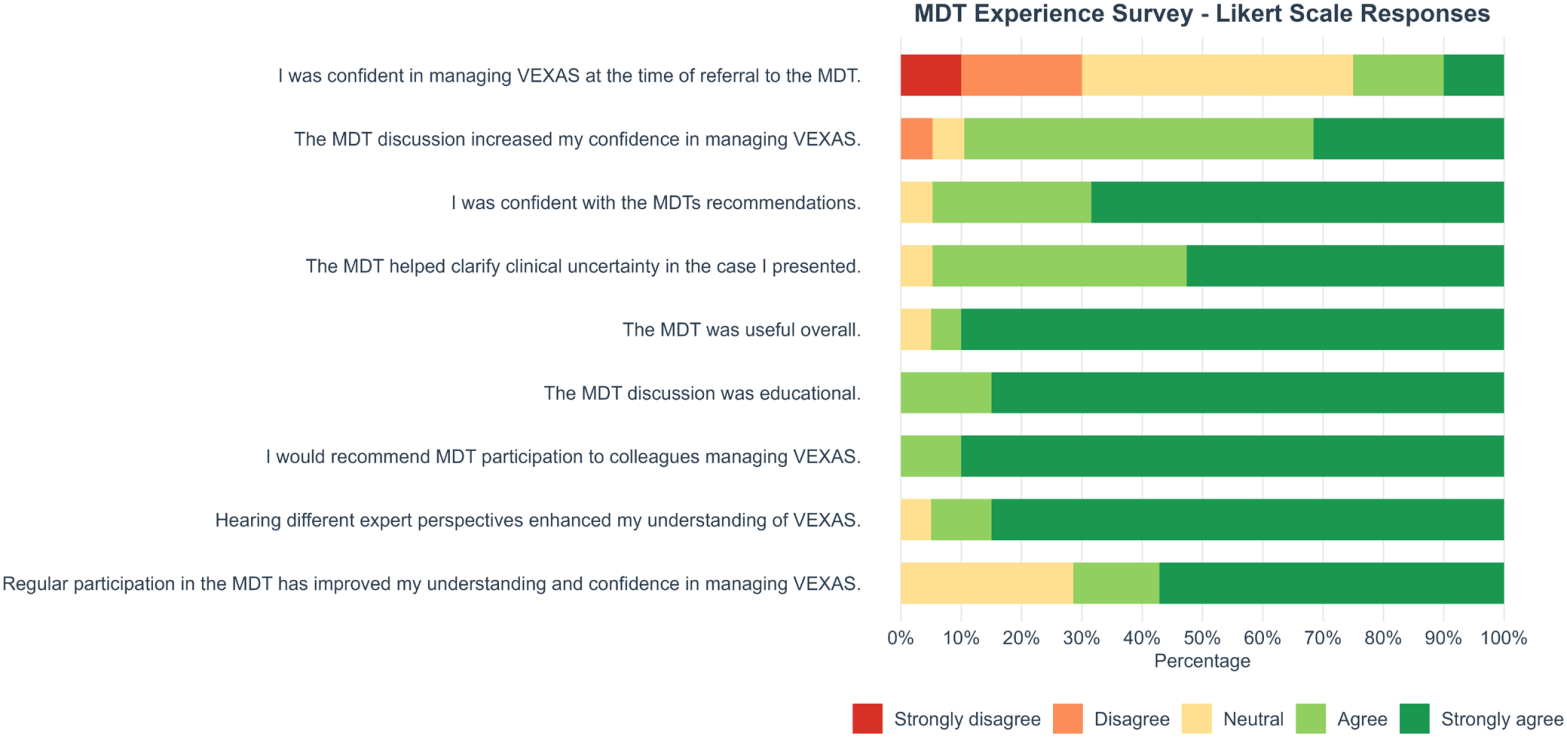
Multidisciplinary team (MDT) experience survey: Responses gave their ratings to each question on a 5‐point Likert scale.

Participants expressed strong agreement with the positive impact of the MDT on managing VEXAS syndrome. The majority agreed that the MDT discussion increased their confidence and helped clarify clinical uncertainties and that they were confident in the MDT's recommendations. Most also found the MDT useful overall and educational and indicated that they would recommend MDT participation to colleagues. Additionally, hearing different expert perspectives and participating regularly in the MDT meetings were reported as having enhanced understanding and confidence in managing VEXAS (see Figure 3).

**FIGURE 4:**
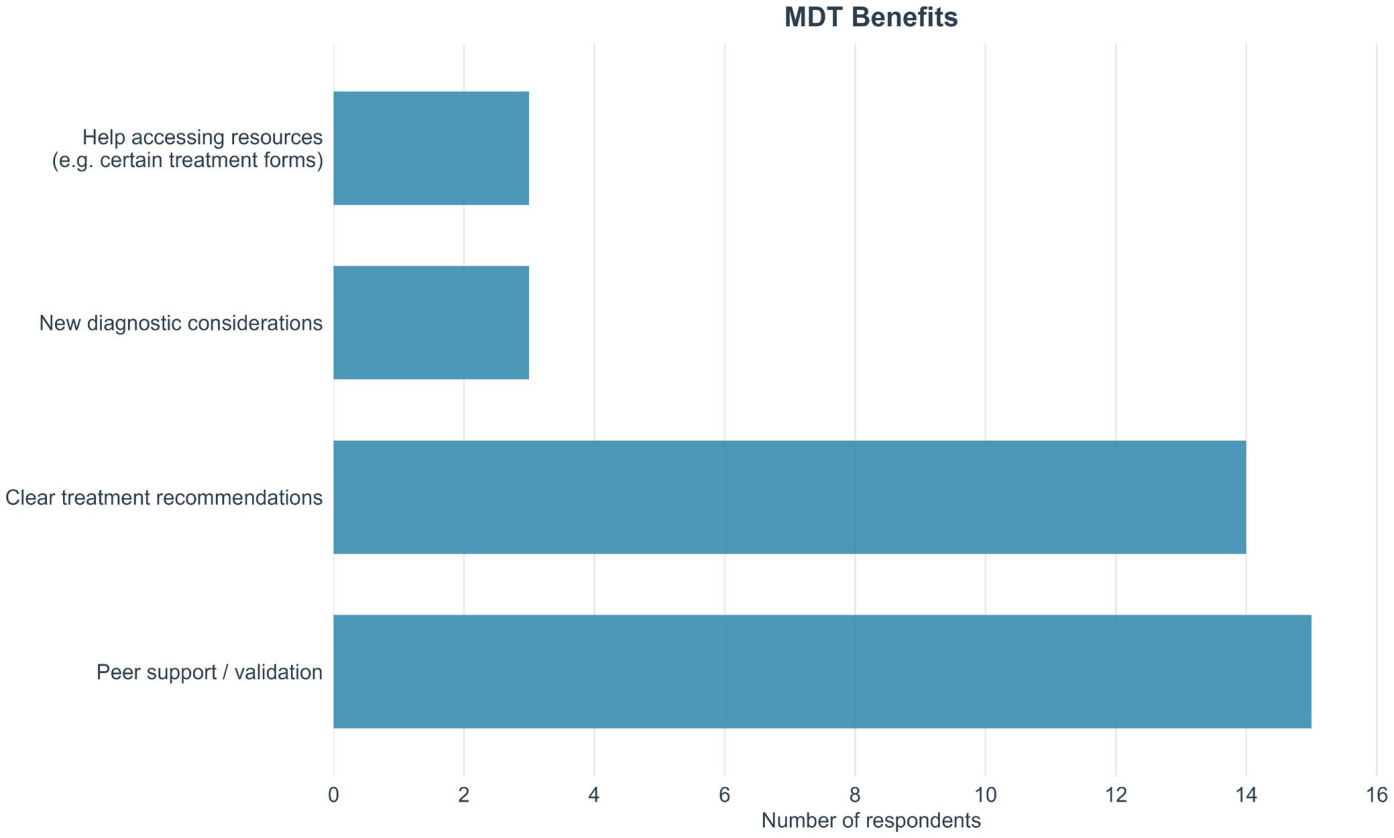
Perceived benefits of the multidisciplinary team (MDT) according to participants.

Most participants identified clear treatment recommendations (*n* = 14) and peer support or validation (*n* = 15) as the main perceived benefits of the MDT (Figure 4). New diagnostic considerations and support in accessing resources—such as treatment options like azacitidine—were mentioned less frequently (each *n* = 3).

Regarding the impact on clinical management, 8 participants reported changing their treatment plan based on the MDT discussion, while 10 did not make changes but felt the MDT confirmed their existing approach.

## DISCUSSION

In this report, we present data from 42 patients with VEXAS syndrome or VEXAS‐like disease who were discussed within a dedicated VEXAS MDT over a 1‐year period, involving 27 centres across the United Kingdom and Ireland. The cohort reflects a broad cross section of real‐world cases seen in routine clinical practice. We also report the results of a survey completed by MDT participants about their experience of participating in MDT meetings.

The high proportion of patients managed with corticosteroids alone, or in combination with methotrexate or other conventional synthetic disease‐modifying anti‐rheumatic drugs (csDMARDs), prior to presentation at an MDT meeting likely reflects two key factors: first, hesitancy to initiate alternative therapies due to limited experience with this newly recognised condition; and second, the fact that many patients initially carried alternative diagnoses, such as seronegative rheumatoid arthritis, prior to their diagnosis of VEXAS syndrome.

Following MDT discussions, there was a clear shift in recommended treatment strategies. Azacitidine and tocilizumab were frequently suggested, consistent with emerging clinical experience.^6^, ^7^, ^9^ In our experience, patients with predominant bone marrow failure appear to benefit most from azacitidine therapy. However, as this treatment is typically outside the scope of non‐haematology specialities, interdisciplinary collaboration is essential, particularly when haematology is not the primary speciality involved in patient care.

In four cases, referral for evaluation for haematopoietic stem cell transplantation (HSCT) was recommended, reflecting its consideration as a potential curative approach in selected patients. Additionally, the observed reduction in csDMARD use after MDT discussions suggests growing consensus regarding their limited efficacy and potential risks in this cohort.^5^ Agents such as methotrexate, for example, carry additional bone marrow suppressive effects, which are particularly unfavourable in patients with VEXAS.^10^, ^11^

Anakinra is generally not considered a first‐line option in VEXAS syndrome due to the high frequency of injection site reactions.^12^ Its recommendation in six cases likely reflects a scenario where other therapeutic options had already been exhausted or where treatment was instituted prior to the discovery of VEXAS syndrome as a disease entity. JAK inhibitors were rarely recommended, which may be attributed to individual prescribing preferences or challenges in accessing specific agents, such as ruxolitinib, despite emerging evidence supporting their efficacy in VEXAS.^6^ This is further complicated by the differential availability and cost of JAK inhibitors within the United Kingdom. Baricitinib, while less expensive and accessible in selected centres, has shown relatively poor clinical effectiveness in the recent UK cohort^7^ potentially limiting clinicians' confidence in its utility for VEXAS syndrome. In contrast, ruxolitinib has demonstrated clearer therapeutic benefit in published case series,^6^ yet its higher cost and more restrictive funding environment significantly reduce its availability. These disparities may contribute to the observed reluctance to adopt JAK inhibitors in routine practice, despite growing international evidence supporting their role in disease control for selected patients. Moreover, with a new clinical trial underway in the United Kingdom, and internationally, the MDT will be a key platform to enable patient access from all regions of the country.^13^

The survey revealed that most participants did not feel confident in managing VEXAS syndrome prior to the MDT, which likely reflects their limited direct experience with affected patients. However, there was a clear consensus on the positive impact of the MDT discussions, particularly in improving participants' confidence and expertise in managing the disease. Notably, there was unanimous agreement regarding the educational value of the MDT.

The fact that peer support and validation were identified as the most valued benefits of the MDT underscores the importance of such collaborative platforms, especially in the context of rare or newly recognised diseases where formal treatment guidelines are lacking. Beyond facilitating knowledge exchange, MDT discussions can also support clinicians with practical aspects of care, including obtaining individual funding for specific therapies and providing medicolegal reassurance by ensuring shared decision‐making in complex cases. Notably, the funding of VEXAS therapies remains a major hurdle in the management of these patients; the absence of licensed treatments and the high cost of off‐label or biological agents often require extensive justification and navigation of variable commissioning processes. As a result, MDT endorsement can play a crucial role in strengthening funding applications and ensuring patients receive timely access to potentially disease‐modifying therapies.

An important limitation of this study is the absence of longitudinal patient outcome data. Our focus was on describing the MDT structure and its immediate impact on treatment decisions, rather than evaluating patient‐level clinical outcomes such as treatment response, steroid‐taper success or survival. As VEXAS syndrome was only described in 2020 and formal guidelines were published during our study period, we prioritised establishing a collaborative framework and assessing its influence on clinical practice. Future work will evaluate long‐term outcomes of MDT‐guided care through systematic follow‐up and potential registry participation.

## CONCLUSION

In summary, our findings highlight the value of interdisciplinary MDT discussions in guiding treatment decisions for VEXAS syndrome, particularly in the absence of management guidelines. The MDT not only facilitates more targeted therapeutic approaches but also provides an important platform for knowledge exchange, peer support and shared decision‐making in this complex and evolving field.

## AUTHOR CONTRIBUTIONS

Daniel Pietsch was responsible for data collection, data analysis and writing the initial draft of the manuscript. Austin Kulasekararaj was responsible for study design and editing of the manuscript. Sinisa Savic was responsible for study design, funding of the study, data analysis and editing of the manuscript. VEXNET‐UK members contributed clinical cases and participated in MDT. All authors read and approved the manuscript.

## FUNDING INFORMATION

SS is funded by Kennedy Trust Senior Fellowship and MRC project grant number MR/Y011945/1. AA, AS, CC and SS are supported in part by the National Institute for Health and Care Research (NIHR) Leeds Biomedical Research Centre (BRC) (NIHR203331). The views expressed are those of the author(s) and not necessarily those of the NHS, the NIHR or the Department of Health and Social Care.

## CONFLICT OF INTEREST STATEMENT

SS has received payments from Novartis and SOBI for advisory board participation and research grant from Novartis. NC has the following COI to declare: Study PI—Novartis, Pharming, Pharvaris, Takeda; advisory boards, lectures—Novartis, Pharming, Takeda, CSL Vifor; conference travel support—Novartis, Pharming, Takeda, Biocryst; unrestricted educational grants—Takeda, Pharming, GSK. AK research support (to institution): Celgene/BMS and Novartis; consultant: Samsung, Novonordisk, Alexion/AstraZeneca; speaker's fees—Alexion/AstraZeneca, Amgen, Celgene/BMS, Pfizer, Novartis, Roche, SOBI, Janssen; scientific advisory board/data monitoring committee—Alexion/Astra Zeneca, Amgen, Agios, Celgene/BMS, Geron, Novartis, Pfizer, Regeneron, Roche, SOBI, Janssen, Samsung, Ascentage Pharma, Takeda, Silence Therapeutics. All other authors have no COI to declare.

## ETHICS STATEMENT

Ethics approval was waived by the local research and ethics committee since this work was done as part of a locally approved audit project using only routinely collected and fully anonymised data.

## Supporting information


Figure S1.

Table S1.

## Data Availability

The datasets generated and/or analysed during the current study are available from the corresponding author on reasonable request. No identifiable or sensitive information is included in the shared materials.
