# Supplementary figures and images for: Navigating through uncertainty—Experience from the UK national VEXAS MDT
Source: Br J Haematol. 2026 Feb 24;208(4):1306–13. doi: 10.1111/bjh.70365 (PMC13071473; doi:10.1111/bjh.70365)

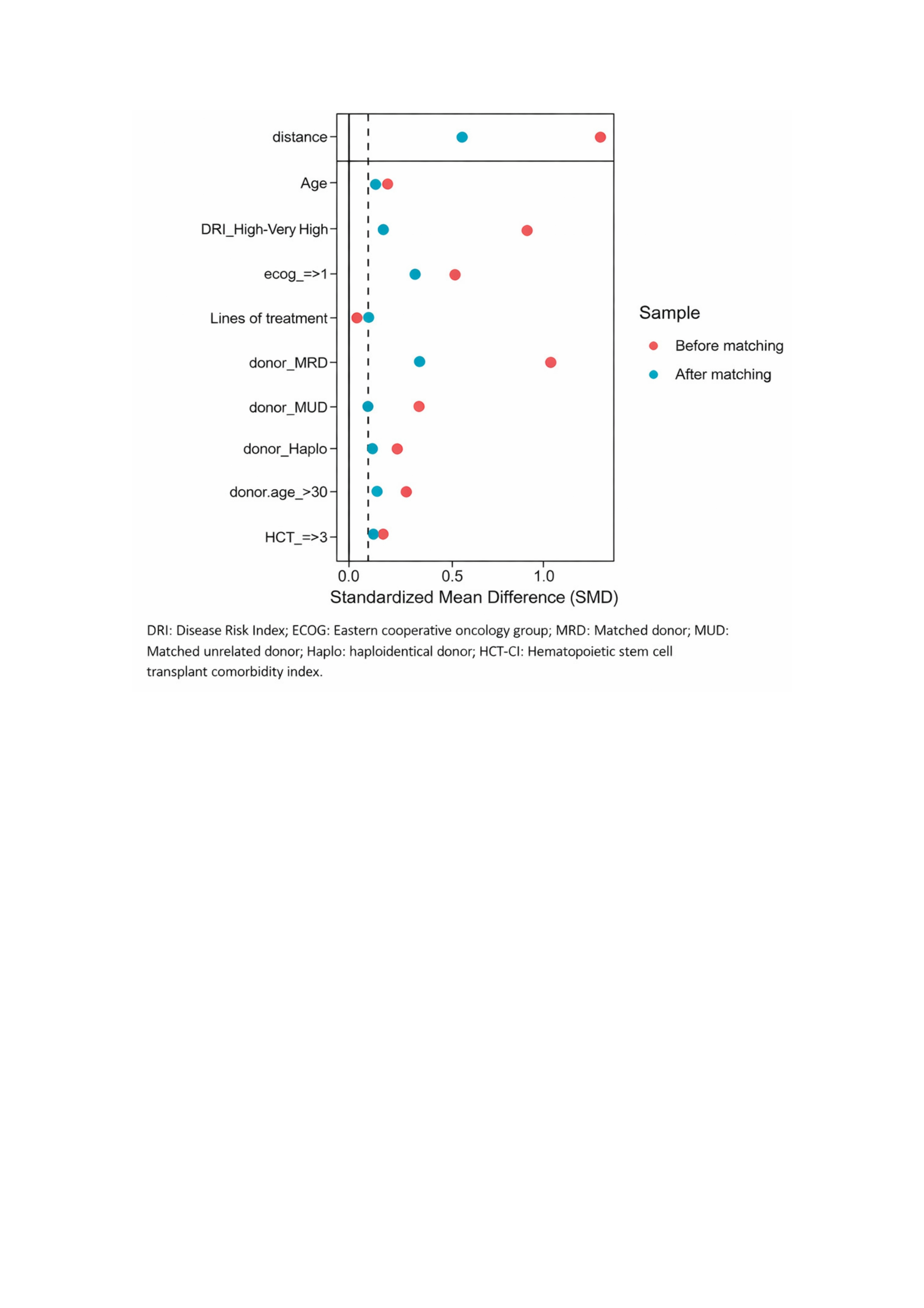

Supplement: Supplementary file 1 — Figure S1. [file BJH-208-1306-s001.jpg]
