# Supplementary material for: Navigating through uncertainty—Experience from the UK national VEXAS MDT
Source: Br J Haematol. 2026 Feb 24;208(4):1306–13. doi: 10.1111/bjh.70365 (PMC13071473; doi:10.1111/bjh.70365)
Supplement: Supplementary file 2 — Table S1. [file BJH-208-1306-s002.docx]

*Supplementary table 1: VEXAS-like patients with no pathogenic variants in UBA1.*

| ID | 1 | 13 | 27 | 35 | 38 | 36 |
| --- | --- | --- | --- | --- | --- | --- |
| Age (years) | 80 | 65 | 79 | 56 | 65 | 44 |
|  | male | Male | Male | Male | female | Male |
| Diagnosis | MDS | MDS | MDS | MDS RCMD | MDS | Pancytopenia of unknown cause |
| Mutations on bone marrow (allele burden) | DNMT3A (21%)  SF3B1 (20%) | TET2, ZRSR2 | IDH1, IDH2, SRSF2, ASXL1 | TET2 (34%) | KRAS, STAG2 | 2x UBA1 VUS |
| Fatigue | + | + | + | - | + | + |
| Fever | + | + | + | + | - | - |
| Rash | - | + | - | + | + | + |
| (Poly-)Chondritis | - | - | - | + | - | - |
| Arthritis/ Arthralgia | + | + | + | + | - | - |
| Respiratory symptoms | + | - | + | - | - | - |
| Transfusion dependency | + | + | - | - | + | - |
| Other |  |  |  |  | Serositis |  |
| Steroid dose* | 30 | 0 | 30 | 30 | 10 | 0 |
| Clinical problem / question | Treatment options for inflammatory symptoms | Steroid-refractory inflammatory features (arthritis, erythematous skin lesions identified as non-necrotizing granulomas on biopsy) | New onset arthritis, raised inflammatory markers | Fevers, skin rash and chondritis despite being on Prednisolone 30mg and Azacitidine; pathogenic MEFV variant | Anemia, skin lesions, raised inflammatory markers; patient rejects Azacitidine  - Diagnostic recommendations | Progressing pancytopenia, scattered blasts on bone marrow, 2 VUS in the UBA1 gene |
| Outcome recommendation | Azacitidine OR Tocilizumab OR Anakinra | Diagnostic recommendations including offer of external review of the biopsies | Review at an external joint clinic with rheumatology/ haematology | Addition of Tocilizumab | Review at a joint clinic with rheumatology/ dermatology/ haematology  Options: Anakinra, JAK inhibitor | Consideration of early bone marrow transplant |

*prednisolone equivalent in mg per day; VUS, variant of unknown significane
